# Sedimentation of large, soluble proteins up to 140 kDa for ^1^H-detected MAS NMR and ^13^C DNP NMR – practical aspects

**DOI:** 10.21203/rs.3.rs-3972885/v1

**Published:** 2024-02-23

**Authors:** Dallas Bell, Florian Lindemann, Lisa Gerland, Hanna Aucharova, Alexander Klein, Daniel Friedrich, Matthias Hiller, Kristof Grohe, Barth van Rossum, Anne Diehl, Jon Hughes, Leonard J. Mueller, Rasmus Linser, Anne-Frances Miller, Hartmut Oschkinat

**Affiliations:** Faculty II-Mathematics and Natural Sciences, Technische Universität Berlin; Leibniz-Forschungsinstitut für Molekulare Pharmakologie; Leibniz-Forschungsinstitut für Molekulare Pharmakologie; Department of Chemistry and Chemical Biology, TU Dortmund University; Department of Chemistry and Chemical Biology, TU Dortmund University; Department of Chemistry and Biochemistry, University of Cologne; Leibniz-Forschungsinstitut für Molekulare Pharmakologie; Bruker Biospin GmbH; Leibniz-Forschungsinstitut für Molekulare Pharmakologie; Leibniz-Forschungsinstitut für Molekulare Pharmakologie; Justus Liebig University, Institute for Plant Physiology; University of California, Riverside; Department of Chemistry and Chemical Biology, TU Dortmund University; Faculty II-Mathematics and Natural Sciences, Technische Universität Berlin; Leibniz-Forschungsinstitut für Molekulare Pharmakologie

**Keywords:** Protein NMR, dense phase, ultracentrifugation, magic-angle-spinning, solid-state NMR

## Abstract

Solution NMR is typically applied to biological systems with molecular weights < 40 kDa whereas magic-angle-spinning (MAS) solid-state NMR traditionally targets very large, oligomeric proteins and complexes exceeding 500 kDa in mass, including fibrils and crystalline protein preparations. Here, we propose that the gap between these size regimes can be filled by the approach presented that enables investigation of large, soluble and fully protonated proteins in the range of 40–140 kDa. As a key step, ultracentrifugation produces a highly concentrated, gel-like state, resembling a dense phase in spontaneous liquid-liquid phase separation (LLPS). By means of three examples, *a Sulfolobus acidocaldarius* bifurcating electron transfer flavoprotein (*Sulf*ETF), tryptophan synthases from *Salmonella typhimurium* (*St*TS) and the dimeric β-subunits from *Pyrococcus furiosus* (*Pf*TrpB), we show that such samples yield well-resolved proton-detected 2D and 3D NMR spectra at 100 kHz MAS without heterogeneous broadening, similar to diluted liquids. Herein, we provide practical guidance on centrifugation conditions and tools, sample behavior, and line widths expected. We demonstrate that the observed chemical shifts correspond to those obtained from μM/low mM solutions or crystalline samples, indicating structural integrity. Nitrogen line widths as low as 20–30 Hz are observed. The presented approach is advantageous for proteins or nucleic acids that cannot be deuterated due to the expression system used, or where relevant protons cannot be re-incorporated after expression in deuterated medium, and it circumvents crystallization. Importantly, it allows the use of low-glycerol buffers in dynamic nuclear polarization (DNP) NMR of proteins as demonstrated with the cyanobacterial phytochrome Cph1.

## Introduction

The majority of proteins and nucleic acids targeted in biomedical research exceed 40 kDa in mass. Their internal dynamics and molecular associations are as important as their static structures, so it is critical to have robust, generally applicable NMR tools that address all three — independently of sample nature and size. Approaches to large proteins include sparse labeling, often of selected residues, that can yield non-overlapping spectra even if samples are composed of so many residues that a full spectrum would be uninterpretable. In other approaches, signals in large proteins are kept narrow *via* exploitation of TROSY effects for methyl group spin systems (Tugarinov et al. 2003; Bolik-Coulon et al. 2023). Furthermore, the incorporation of fluorinated aromatic amino acids provides selectivity and exploits the enlarged dispersion of ^19^F chemical shifts (Huestis and Raftery 1975; Luck and Falke 1991; Gronenborn 2022). However, these approaches are restrictive regarding amenable residues. Thus, methodologies based on heteronuclear correlations, suitable for all amino acids but potentially in conjunction with amino-acid selective labeling, are more desirable.

**Figure 1:**
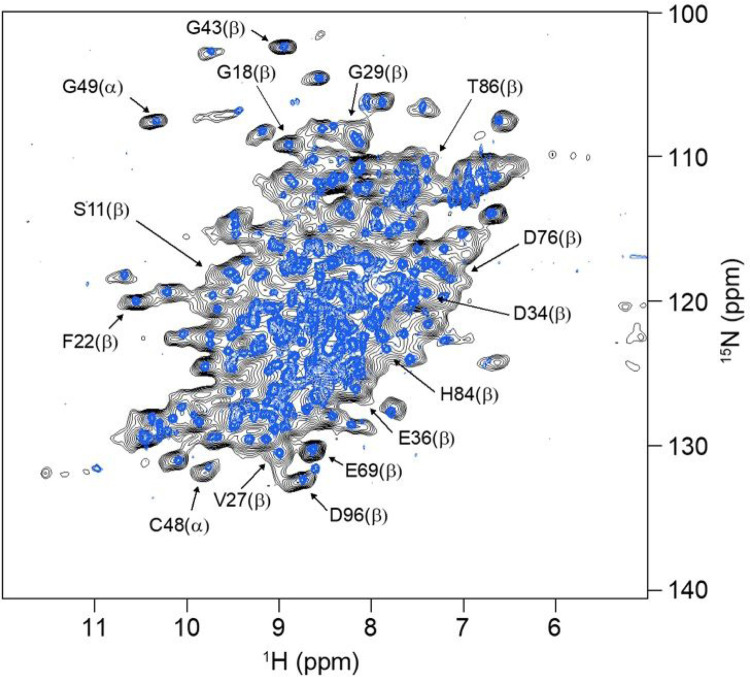
^1^H-^15^N NMR correlations of the ^13^C, ^15^N-labelled extracellular domain of human neonatal Fc receptor recorded in solution (blue spectrum) using an HSQC pulse sequence and at 100 kHz MAS (black spectrum) by cross polarization. Examples of resonance assignments based on triple-resonance NMR experiments are indicated for the a-chain (a) and the b2-microglubulin (b) subunits of the heterodimeric extracellular domain of receptor (Stöppler et al. 2018).

A suitable perspective is given by MAS of soluble proteins after sedimentation, first applied to obtain resolved NMR spectra of very large oligomeric systems (> 300 kDa) such as αB-crystallin (Mainz et al. 2009) and α-Ferritin (Bertini et al. 2011, 2012, 2013). Subsequent experiments with concentrated human superoxide dismutase showed that this approach works also for smaller proteins (Fragai et al. 2013). Later, Stöppler et al. demonstrated the potential of the approach for structural investigations of small proteins by sedimenting the fully protonated, 42 kDa extracellular unit of the neonatal Fc-receptor (Stöppler et al. 2018). Without deuteration at the non-exchangeable sites, well-resolved ^1^H-^15^N correlations (Bodenhausen and Ruben 1980) were obtained *via* cross polarization (Paulson et al. 2003; Zhou and Rienstra 2008) after concentrating, effectively sedimenting the protein into the MAS NMR rotor by ultracentrifugation. Sets of 3D NMR spectra suitable for achieving resonance assignments could be obtained at 100 kHz MAS. The protein’s structural integrity and thus the validity of the approach were demonstrated by the similarity of the ^1^H-^15^N INEPT-based HSQC recorded on a μM/low mM solution, henceforth called ‘dilute’, with the corresponding MAS NMR spectrum recorded with a CP-based sequence (Fig. 1). Moreover, this investigation provides proof-of-principle for an alternative approach to the investigation of large proteins that is independent of suitably labeled amino acid types or special spectroscopic effects. Most commonly, compensating relaxation pathways within certain types of spin systems such as methyl groups are exploited.

In a study on the longevity of MAS NMR samples containing sedimented protein together with small ligands and oligonucleotides, Wiegand et al. applied this concept to a set of proteins or their subunits comprising a bacterial helicase, a polymerase, a primase and an ABC transporter, by employing ^13^C-detection in 3.2 mm rotors (Wiegand et al. 2020). In all these cases, the complexes remained intact, as no signs of sample heterogeneity were detected after several years of storage in rotors and repeated MAS NMR experiments. Yet, a layer of gel-like sample was found at the rotor walls, indicating that the gel-like solution present in the rotor prior to experiments had changed to an even denser phase.

Besides the beneficial increase of protein concentration by ultracentrifugation with retention of solution-like spectral properties, the high protein concentration is advantageous for the needs of MAS DNP NMR (Hall et al. 1997; Barnes et al. 2008). In such experiments, concentration of the polarizing agent during freezing must be avoided to ensure long lifetimes of electron spin states and thus NMR signal enhancements. Usually, this is achieved by adding significant amounts of glycerol to the DNP sample to ensure that a glass-like state is obtained upon freezing (Hall et al. 1997). Since a reduction of the glycerol content might be advantageous in individual cases such as in investigations of membrane proteins or protein-cofactor complexes, we tested our approach with the cyanobacterial phytochrome 1 (Cph1) from *Synechocystis* that contains the cofactor phycocyanobilin (PCB).

In the current work, we demonstrate that MAS-responsive samples of proteins in the 40–140 kDa range can be generated in a systematic manner by an ultracentrifugation procedure. Even for non-deuterated proteins, resolved ^1^H-^15^N correlations and 3D spectra are obtained at 100–110 kHz MAS from the produced dense phase. With the example of the *Sulf*ETF we demonstrate that a nitrogen line width in the 20–30 Hz range can be achieved, and sample lifetimes extend over many weeks. By means of the two tryptophan synthase samples (tetrameric and β-subunit dimers) we demonstrate sample stability and sensitivity, enabling the acquisition of data sets for resonance assignments and distance restraints. In all cases, we provide evidence of close similarity of the ^1^H-^15^N correlations recorded on sedimented samples with those obtained from dilute solutions or from crystals as a proof of sample integrity. Importantly, more complete spectra than by solution NMR of deuterated proteins are obtained in which signals are often missing due to insufficient back-exchange of amide protons. Furthermore, we demonstrate by means of the example of the phytochrome Cph1 that this approach is suitable for minimizing freezing-associated formation of ice crystals in DNP samples with very low levels of glycerol. In the case of the phytochrome Cph1 we demonstrate that the glycerol content of the sample could be reduced to about 10%. The procedure ensures substantial signal enhancements attainable via DNP at 800 MHz ^1^H NMR frequency.

## Materials and methods

### Sulf ETF

*Sulf*ETF was expressed from a codon-optimized version of the gene AHC50813.1 (GenBank) synthesized by GenArt Germany and cloned into a pET-derived vector, by using a modular cloning kit (similar to golden gate assembly). The plasmid was transformed into *E. coli* BL21(DE3) cells selected with kanamycin. Expression was induced using isopropyl β-D-thiogalactopyranoside (IPTG) at OD_600_ 0.8. After 18 further hours at 18°C, cells were harvested, washed and frozen at −80°C until needed. For uniformly labeled ^15^N-*Sulf*ETF, the construct was expressed in M9 medium, including 2 g ^15^NH_4_Cl / L culture. Protein purification employed Ni-NTA affinity chromatography. Oligomers were removed by Superdex200 size exclusion chromatography column and the protein subsequently stored in buffer comprising 20 mM TRIS/HCl, 100 mM KCl at pH 7.8. The same buffer was used for the solution NMR measurements after adding 10% D_2_O. For solid-state NMR measurements, the ^15^N-labelled protein was sedimented into a 0.7 mm ZrO_2_ rotor as specified in Table 1. All measurements were conducted on a Bruker Avance NEO spectrometer operating at 900 MHz ^1^H Larmor frequency. Water suppression was achieved with the MISSISSIPPI sequence for solid-state NMR and the WATERGATE sequence in solution. Decoupling was always achieved by WALTZ16 (^1^H, ^13^C, ^15^N). All solid-state NMR spectra were acquired at 100 kHz MAS. A detailed listing of experimental parameters can be found in Tables S1, S2, and S3.

The water content of the sample was estimated on the basis of the integrated 1D ^1^H NMR spectra shown in Fig. S5. The integral of the region between 4.2 and 6 ppm was considered to reflect largely water, and the areas to the right and left protons of the protein. Protein signals under the water lead to an error, yet the water line had a hump extending towards 3 ppm, leading to an error in the opposite direction. We assumed a compensation of both. For the fresh sample, the integral of the water area is 3.8 times larger than the sum of the other two that are reflecting the protein. Since we are interested in the water/protein ratio, it is fair to assume that the protein integral reflects 4862 protons (for one molecule), and the water signal contains 2 protons times × molecules. With an integral ratio of 3.8 and molecular weights of 66843 and 18 a 2.5-fold excess of water (w/w) is obtained.

## Tryptophan Synthase

Protein expression was as described in (Caulkins et al. 2016). The ^13^C, ^15^N-labeled protein was kept in 50 mM Caesium bicine buffer, pH 7.8, in the presence of 3 mM N-(4′-trifluoromethoxybenzenesulfonyl)-2-aminoethyl phosphate (usually termed F9; a high affinity alpha site ligand). 50 μL of 20 mg/mL protein were sedimented into a 0.7 mm ZrO_2_ rotor with the conditions specified in Table 1. The NMR measurements were conducted on a Bruker Avance NEO spectrometer operating at 900 MHz ^1^H Larmor frequency and at 100 kHz MAS. Water suppression was achieved with the MISSISSIPPI sequence and decoupling by slTPPM (^1^H) and WALTZ16 (^13^C, ^15^N). A detailed overview of experimental NMR parameters can be found in Tables S4 and S5.

### Pf TrpB

Samples of *Pf*TrpB (measurements in 0.7 mm rotors) and *Pf*TrpB(2B9) (1.3 mm rotors) were prepared with the following protocol. For the *Pf*TrpB sample, trivial modifications were employed (protonated, ^13^C-labeled glucose vs. deuterated, ^13^C-labeled glucose, and fewer cycles of media inoculation). The gene encoding *Pf*TrpB(2B9), containing the mutations I16V, E17G, I68V, F95L, F274S, T292S, T321A, V384A (UNIPROT ID Q8U093) was previously codon-optimized for *E. coli* and cloned into a pET22(b) + vector with an uncleavable C-terminal 6xHis tag. A single colony of *E.coli* BL21 (DE3) was used to inoculate a 5-mL culture of LB media with 100 μg/mL carbenicillin (LBcarb) and incubated overnight at 37°C at 180 rpm. This culture was used to inoculate a 50 ml LBcarb culture, which was incubated at 180 rpm and 37 C for 4 hours. Then, 500 μl of culture was used to inoculate 50 ml of modified M9 media dissolved in water (M9mod/H_2_O) and supplemented with carbenicillin, and was incubated at 180 rpm and 37°C for 4 hours. After that, 500 μl of culture was used to inoculate 50 ml of a composite minimal medium mixture comprising 10% M9mod/D_2_O and 90% M9mod/H_2_O, supplemented with antibiotic, and incubated at 180 rpm and 37°C overnight. Next morning, 500 μl of culture was used to inoculate 50 ml of 50% M9mod/D_2_O and 50% M9mod/H_2_O and incubated at 180 rpm and 37 C for 4 hours. Finally, 500 μl of a resultant culture was transferred into a 5 L flask containing 2 L of M9mod/D_2_O medium supplemented with carbenicillin, and incubated at 180 rpm and 37°C until OD_600_ reached 1. Cultures were chilled on ice for 20–30 min and expression was induced by the addition of IPTG to a final concentration of 1 mM. Cells continued to grow shaking at 180 rpm and 20 C for another 20 h. Cells were harvested by centrifugation at 4 C and 4,000 g for 20 min; the pellets were frozen at −80 C until further use.

Frozen cell pellets were thawed at room temperature and resuspended in buffer A (50 mM phosphate buffer, pH 8.0, with 20 mM imidazole and 100 mM NaCl) supplemented with 200 μM PLP, 1 mM PMSF, 1mM TCEP, 1 mg/mL hen egg white lysozyme, and 0.02 mg/mL DNAse. After vortexing, cells were lysed with an Emulsiflex C3 (three runs for each culture). Lysates were aliquoted into 45 ml tubes and centrifuged at 100,000 g and 4°C for 20 min, then incubated at 75°C for 10 min and spun again. The clarified, heat-treated lysate was applied to a 5 ml Ni-NTA column. The protein was eluted by gravity with buffer B (50 mM phosphate buffer pH 8.0, 500 mM Imidazole, 100 mM NaCl). Appropriate fractions were combined and exchanged into NMR buffer (50 mM phosphate buffer pH 8.0, 1mM TCEP) using a PD10 column, frozen in liquid N_2_, and stored at −80 C until further use.

For sedimentation into a 0.7 rotor, the protein was produced in iFD-labelling (Medeiros-Silva et al. 2016) fashion (using deuterated and ^13^C-labelled glucose, ^15^N-labelled NH_4_Cl, and H_2_O as a solvent) and spun for 94 h in a Beckmann SW 40 Ti rotor at 20,000 rpm (71,100 g) and 20°C. For sedimentation into a 1.3 mm rotor, protein was produced in triple-labelled fashion (deuterated and ^13^C-labelled glucose, ^15^N-labelled NH_4_Cl, and D_2_O as a solvent) and sedimented for 72 h at 210,000 g and 20°C.

Solid-state experiments for determining ^1^H bulk sensitivities were recorded on a Bruker Avance NEO 700 MHz (^1^H Larmor frequency) spectrometer either using a 1.3 mm rotor at an MAS frequency of 55.5 kHz or using an 0.7 mm rotor at 100 kHz MAS. Experiments were recorded using 256 scans to obtain an adequate signal-to-noise ratio (S/N) with a recycle delay 1.3-fold the measured T_1_ times (^1^HN bulk) (see also caption of Fig. S9). The intensities represent the first FID of each nD experiment, i.e. using a 200 ns delay for each t_n_/2 period and including all necessary pulses for (selective) decoupling. The S/N was determined in Topspin using the same signal and noise region for a given sample. A detailed listing experimental parameters can be found in Tables S6–S9.

### Cyanobacterial phytochrome Cph1 from Synechocystis

The plasmid encoding Cph1Δ2 (the N-terminal NTS-PAS-GAF and PHY domains of Cph1 with a C-terminal H_6_ tag) was cotransformed with pSE111 into *E.coli* BL21 DE3 cells using a standard heat shock protocol. A 30 ml preculture was grown for 1h at 37°C, 170 rpm on LB medium containing kanamycin (40 μg mL^− 1^) and carbenicillin (60 μg mL^− 1^). Cells were spun down (RT, 10 min, 2000 g) and resuspended in 15 mL 2x M9 medium (D_2_O, Glucose, NH_4_Cl). Cells were grown overnight at 32° C, 170 rpm. Glucose and NH_4_Cl were added and cells kept at 37° C for 1h. Then, after reaching an OD_600_ of 4.7 (after about 1h at 18°C, 170 rpm), protein expression was induced with 100 μM IPTG. During expression, Glucose and NH_4_Cl were added when glucose and ammonium chloride levels approached zero. After 48 h cell were harvested at 18°C, 160 rpm, washed in 150 mM NaCl and stored at −80°C. For the final steps such as binding of PCB chromophore and photoconversion into the PR-form standard protocols were applied (Essen et al. 2008; Hughes 2010). A non-protein labelled Cph1Δ2 sample was assembled with ^1^H, ^13^C and ^15^N-labelled PCB. 15 mg of Cph1Δ2 in 50 mM Na/PO_4_ buffer (pH 7.8, 20% D_2_O) were filled into a 1.9 rotor via ultracentrifugation for 68 h, 4°C, 71,000 × g.

1D hC-CP spectra were recorded with 1536 complex points, 16324 scans, an ^13^C acquisition time of 15 ms and a spectral width of 81.5 kHz. For CP, constant ^1^H rf-field of 80 kHz was applied while the ^13^C spin-lock pulse was ramped from 40 to 50 kHz. The CP contact time was set to 2000 μs. During acquisition 100 kHz Spinal64 proton decoupling was applied. The recycle delay was set to 2 ms.

## Results and discussion

### The preparation procedure

**Figure 2:**
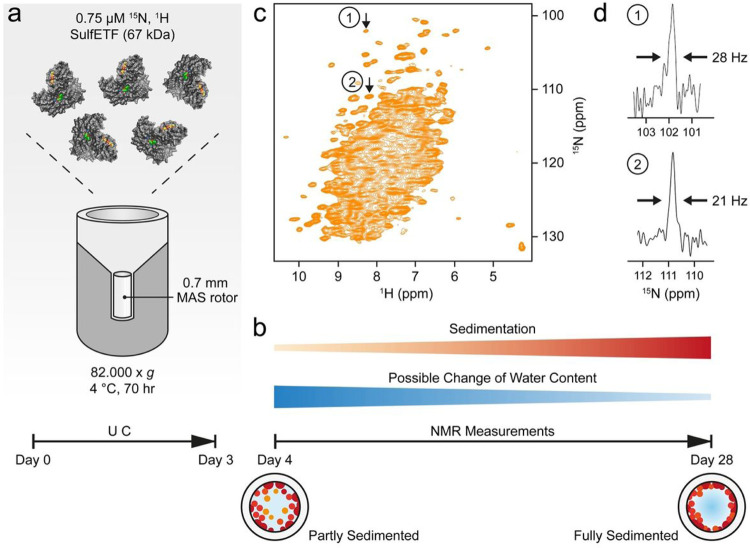
Procedure for the preparation of sedimented samples. (a) Ultracentrifugation (UC) of the sample directly into the rotor using the tool shown in Fig. S1 and S2. (b) Development of the sample during measurements. (c) ^1^H-^15^N NMR correlation of ^13^C/^15^N-labelled *Sulf*ETF recorded at 100 kHz MAS, 294 K, and a proton frequency of 900 MHz. (d) Slices from a similar spectrum as in (c) but recorded with 2048 t_1_ increments for a spectral width of 142 ppm in the nitrogen dimension, taken at the indicated cross peak positions 1 and 2 along F_1_(^15^N).

The quality of the NMR measurements described below is critically dependent on the sample preparation protocol that produces a dense phase by ultracentrifugation (Table 1, Fig. 2). In general, the protein is concentrated by centrifugation at 70 000–85 000 g for up to 2–3 days, depending on the protein. The protein is either directly spun into the rotor (Fig. 2a), or centrifuged into a rotor after concentration of the protein in a separate centrifugation step from which only the concentrated phase is retained. When using small rotors (e.g., 0.7 mm), a filling tool is required for which the engineering drawings are shown in Figs. S1 and S2. A 0.7 mm rotor can contain up to 600 nL of material, whereas the funnel of the filling tool accommodates 250 μL (Fig. S2), so loading the funnel with an initial, low concentration of 1–4 mg/ml protein yields sufficient volume and concentration in the rotor after centrifugation. Table 1 lists the conditions applied to prepare the samples mentioned in this work. The conditions may also be applied to fill rotors with other diameters, using an adapted filling tool.

In case of *Sulf*ETF (Fig. 2a) the ratio of protein-to-water approached 1:2.5 (w/w) (Fig. S6). At that point, the sample is gel-like, often not amenable to transfer by pipetting and does not redissolve into the supernatant. It strongly resembles a dense phase observed in LLPS (Alberti and Hyman 2021). During NMR experiments under MAS, the protein is centrifuged further to the rotor wall (Fig. 2b) due to the much higher g-force experienced than in the ultracentrifuge. After the measurements, we opened a rotor containing StTS to assess the condition of the sample. A notable amount of gel was apparent on the rotor walls, as shown in Fig. S3. Nevertheless, the spectra obtained from this rotor did not change over a period of months, indicating that this state forms quickly at the start of the measurements through the effects of centrifugation.

Cross-polarization is now effective and ^1^H-^15^N correlation spectra with narrow lines are obtained (Fig. 2c). Importantly, no signs of an amorphous state or fibrils are observed. Care must be taken to reduce water evaporation during spinning at the given temperature: rotor caps must either be tight enough, be glued into the rotor or be fitted with sealing plugs. High-quality spectra can then be acquired for at least two weeks, but usually much longer if water loss is prevented.

#### Sulfolobus acidocaldarius ETF

Many redox-active enzymes employ conformational changes to regulate electron transfer in accordance with substrate availability and access to partner proteins (Rees and Howard 1999; Huang et al. 2013). Moderately simple examples are electron transfer flavoproteins (ETFs), one of which mediates electron bifurcation in anaerobes, including archaea (Peters et al. 2016; Garcia Costas et al. 2017; Duan et al. 2018; Müller et al. 2018). *Sulf*ETF has 609 amino acids and a molar mass of 67 kDa. It contains two flavin-adenine-dinucleotide (FAD) molecules, one bound in each of the two domains: one 14 kDa domain, termed the ‘head’, and another 53 kDa ‘base’ domain. In an attempt to understand structural rearrangements as a response to catalysis, which is expected to involve an 80° rotation of its head domain (Toogood et al. 2005; Demmer et al. 2017; Murray et al. 2024), we plan to characterize structure and dynamics of this system by MAS NMR in further studies.

**Figure 3:**
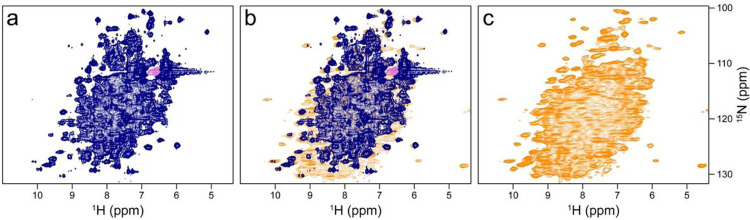
Comparison of the CP-based ^1^H-^15^N NMR correlation of non-deuterated *Sulf*ETF shown in Fig. 2 c with a corresponding solution NMR spectrum of a sample deuterated in the non-exchangeable sites. Both spectra were recorded at 900 MHz Larmor frequency. (a) ^1^H-^15^N HSQC of a dilute solution of ^2^H, ^15^N-labelled *Sulf*ETF (b) Overlay with the ^1^H-^15^N NMR correlation shown in Fig.2 c. (c) Spectrum shown in Fig. 2c, for comparison.

The sedimented samples yielded a well-resolved ^1^H-^15^N correlation (Fig. 2c, d) by a CP-based pulse sequence (Paulson et al. 2003; Zhou and Rienstra 2008) at 100 kHz MS, whose dispersed cross peaks overlay well with those of an INEPT-based ^1^H-^15^N HSQC spectrum (Bodenhausen and Ruben 1980) recorded on a deuterated sample (Fig. 3a). The advantage of the CP-based approach is evident from the overlay (Fig. 3b), in that it visualizes numerous signals that are not evident in the ^1^H-^15^N INEPT HSQC spectrum of the deuterated sample. This is most apparent from slices along F_1_ taken at 8.95 ppm ^1^H chemical shift as shown in Fig. S4. Most likely, these signals with chemical shifts characteristic of β-sheet structure are missing in the solution NMR spectrum owing to insufficient exchange of protons into backbone positions in *Sulf*ETF expressed in 100% D_2_O-containing medium.

Overall, the MAS NMR spectrum shows a large number of dispersed cross peaks that permit measurement of the line widths. In a CP-based ^1^H-^15^N correlation recorded with a spectral resolution of 12.6 Hz in t_1_ (2048 t_1_ increments for 142 ppm), we observe a width at half-height of 20–30 Hz for nitrogen signals when processed without weighting functions (Fig. 2d, see also arrows in 2D spectrum, Fig. 2c). In the same spectrum, proton linewidths in the range of 150–300 Hz are observed. Minimal water loss occurred during the initial measurement sessions, as indicated by a comparison of 1D ^1^H spectra, recorded on the fresh sample and one year later (Fig. S5). These spectra revealed that the initial protein-to-water ratio of approximately 1:2.5 (w/w) only changed marginally over this period. Furthermore, the state of the sample remained largely the same, as indicated by a CP correlation recorded after one year (Fig. S6).

**Figure 4:**
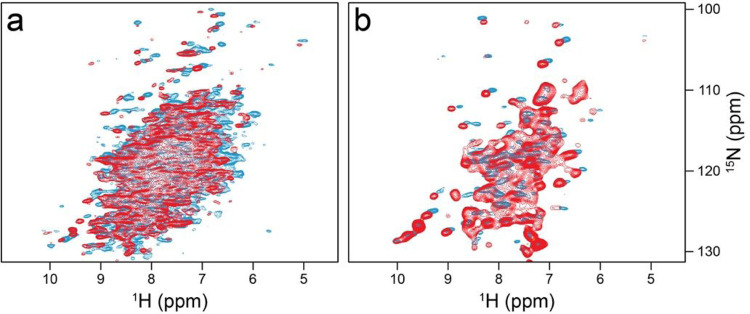
Comparison of CP-based (a) and INEPT-based (b) ^1^H-^15^N correlations of *Sulf*ETF recorded at 289 and 313 K (blue and red, respectively), both under 100 kHz MAS and at 900 MHz proton Larmor frequency.

There is a striking difference in peak pattern between the CP-based (Fig. 4a) and INEPT (Fig. 4b) ^1^H-^15^N correlations, which indicates a residual overall motion of the protein (Aebischer and Ernst 2024). The INEPT spectrum shows only a subset of peaks with mostly random-coil chemical shifts, while the CP-based correlation gives the impression of a large protein with the expected dispersion. CP signal intensities were stronger at lower temperatures (see Fig. 4, blue 289 K and red 313 K). The high quality of the two spectra indicates that the protein molecules do not show the isotropic tumbling behavior observed in dilute solution, where transfers mediated by dipolar couplings are largely compromised. On the other hand, if the system were completely rigid, the T_2_ times of all proton and nitrogen signals would be long enough at fast MAS to show the same cross peak patterns in Fig. 4b than those observed in Fig. 4a. In this case, a complete ^1^H-^15^N correlation would be expected *via* INEPT transfer. There is obviously considerable residual motion, in line with the observed absence of heterogeneous line broadening.

### Tryptophan synthase

*St*TS consists of two α (268 amino acids) and two β (397 amino acids) subunits. They assemble into an αββα heterodimer with a total molecular weight of 144 kDa. While 126 X-ray structures are available in the PDB at the time of writing, knowledge of local mobility and conformational transitions, as well as the protonation states (particularly within the active site) (Caulkins et al. 2016; Holmes et al. 2022), is required to understand the catalytic cycle of the protein. Such factors are accessible by NMR after assigning chemical shifts. In addition to the four-subunit StTS, we also investigated isolated β-subunit dimers (*PfT*rpB, with a total molecular weight of 85 kDa, and the mutant *PfT*rpB(2B9)). To estimate the perspectives of obtaining resonance assignments on sedimented samples in each case, we recorded a set of representative 3D data sets for *St*TS as well as the rst scans of a series of different triple-resonance experiments for *Pf*TrpB.

**Figure 5:**
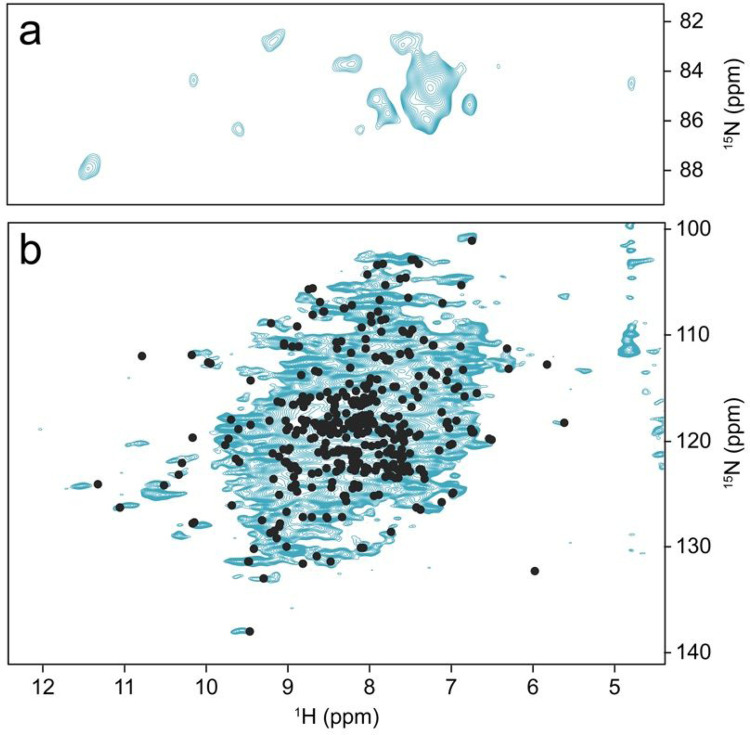
2D CP-based ^1^H-^15^N NMR correlation recorded on fully protonated and ^13^C, ^15^N-labelled *St*TS in a 0.7 mm rotor at 100 kHz MAS. (a) Arginine NeH region of the spectrum. (b) Amide NH region.

We first sedimented ^13^C, ^15^N-labeled StTS into a 0.7 mm rotor as described above and measured a CP-based ^1^H-^15^N correlation of which the NH region of the arginine residues is shown in Fig. 5a and the amide signal region in Fig. 5b. The resonance assignments previously obtained from a crystalline sample (Klein et al. 2022) are indicated by dots. Those assignments match for cross peaks with chemical shifts separate from the bulk signal conglomerate, which are the ones most affected by changes in local environment (δ ^1^H > 9.7 ppm; δ ^15^N > 130 ppm). Thus, the sedimented protein very likely retains the same structure as revealed by X-ray crystallography (Niks et al. 2013) and by NMR on a crystalline sample (Klein et al. 2022).

As in the example before, we note more signals in the spectral region characteristic of β-sheets, between 9.5 and 10.5 ppm ^1^H chemical shift, than there are assignments indicated. Their most likely origins are the regions of the TIM barrel (Wu et al. 2007) buried inside the α subunit that were previously unassigned. In order to test the feasibility of acquiring data sets suitable for resonance assignment, we recorded 3D hCONH and hCANH spectra (Fig. S7a, b for three-dimensional view and Fig. S7c, d for ^1^H-^13^C projections) (Barbet-Massin et al. 2014). The ^1^H-^15^N projections of the hCONH and hCANH spectra (Fig. S7e, f) match the 2D ^1^H-^15^N correlation. The two spectra each contain only one set of signals, indicating sample stability over the time of measurement. Altogether, this opens a different, more comprehensive avenue for sequence-specific resonance assignments and collection of structural constraints. As an additional advantage of fully protonated samples investigated at 100 kHz MAS, the CP-based ^1^H-^13^C correlation (Fig. S8) provides access to ^1^H chemical shifts of the side chains.

**Figure 6:**
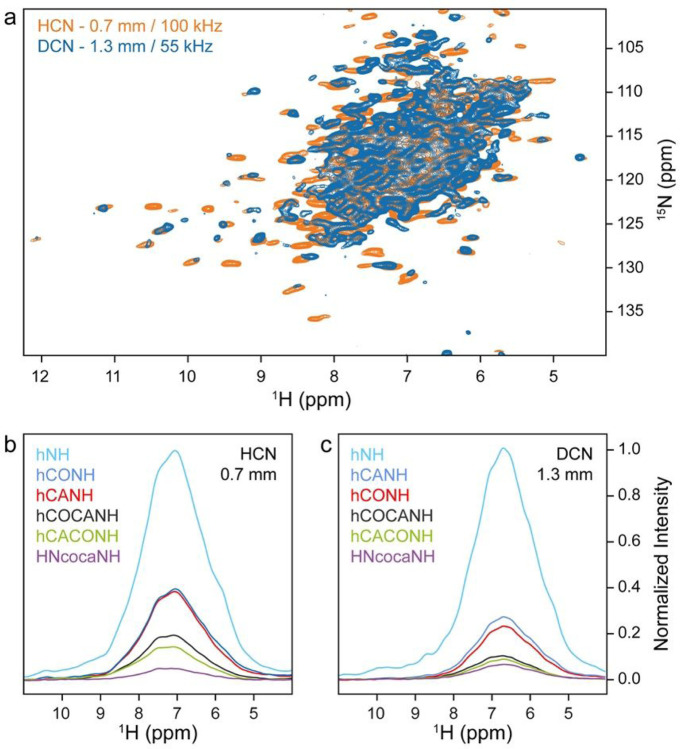
Comparison of spectra recorded on sedimented *Pf*TrpB and *Pf*TrpB(2B9) (~85 kDa) at 700 MHz proton Larmor frequency. (a) Superposition of 2D ^1^H-^15^N NMR correlations of iFD-labeled *Pf*TrpB in a 0.7 mm rotor (yellow) and of *Pf*TrpB(2B9) fully deuterated at the non-exchangeable sites in a 1.3 mm rotor (blue), both sedimented under comparable conditions. (b) Ratios of bulk intensities obtained from the first increment of the indicated 2D, 3D or higher-dimensionality experiments recorded at 100 kHz. (c) The same for the deuterated sample in a 1.3 mm rotor spinning at 55 kHz. Sequences and parameters used are similar to Klein et al. (Klein et al. 2022). For parameters used see SI.

*St*TS is already quite large, making ultracentrifugation and MAS effective. To test the performance of pulse sequences that deliver a data set for achieving assignments on a smaller protein, we employed the *Pf*TrpB sample comprising β-subunit dimers. In this case, the protein was uniformly ^13^C, ^15^N-labeled and the protonation content tuned according to the iFD protocol (H_2_O-based medium with deuterated glucose) to assure full protonation at amide sites while introducing a high degree of side chain deuteration (Medeiros-Silva et al. 2016). Again, a well-resolved spectrum is obtained at 100 kHz MAS, with additional cross peaks observed in comparison to the spectrum of a perdeuterated sample (*Pf*TrpB(2B9)) recorded at 55 kHz MAS (Fig. 6a). Remarkably, the S/N of both samples was not too different, bearing in mind, however, that probably the 1.3 mm rotor was not completely filled and that a generalizable quantitative comparison of samples with different labelling pattern investigated in rotors with different diameters and using different probe heads is not possible. We thus recorded for both b-subunit samples 1D bulk amide proton signals from hNH, hCANH, hCONH, hCOCANH, hCACONH, and HNcocaNH experiments (Klein et al. 2022), and compared bulk intensities within the sets (Fig. 6b and c). Relative to the hNH experiment, we obtained more intense signals for the 3D sequences in case of the iFD sample in the 0.7mm rotor at 100 kHz MAS. For separate plots with percentages see Fig. S9.

A special opportunity for studying protein function is offered by the presence of arginine head group signals in a characteristic region of the ^1^H-^15^N NMR correlation (Fig. 5a). In the case of *St*TS, R141 of the β-subunits is critically important for activity. It plays an essential role in stabilizing the closed form of the subunit by forming a salt bridge with D305 upon substate binding. Furthermore, it is part of the larger COMM domain (β102-β189) involved in the allosteric regulation of the catalytic activities of the α- and β-subunits (Schneider et al. 1998; Ghosh et al. 2022). The corresponding side chain signals may be resolved in this less crowded area of the spectrum and assigned by mutation.

### Phytochrome Cph1 and a path to low-glycerol DNP samples

DNP NMR samples of the sensory module of the cyanobacterial phytochrome Cph1 (Rockwell et al. 2006; Essen et al. 2008; Hughes 2010) were prepared for two purposes: (i) to investigate potential exchange broadening of its chromophore carbon signals in measurements at room temperature that could occur analogously to previously detected amide ^15^N signal broadening (Hahn et al. 2008) and (ii) to assess the quality of DNP NMR samples prepared with minimal glycerol content. In both cases, the light-sensitive protein was investigated in 1.9 mm rotors. Here, the preparation of a gel-like phase was seen as an opportunity to reduce the amount of glycerol in DNP NMR samples.

**Figure 7:**
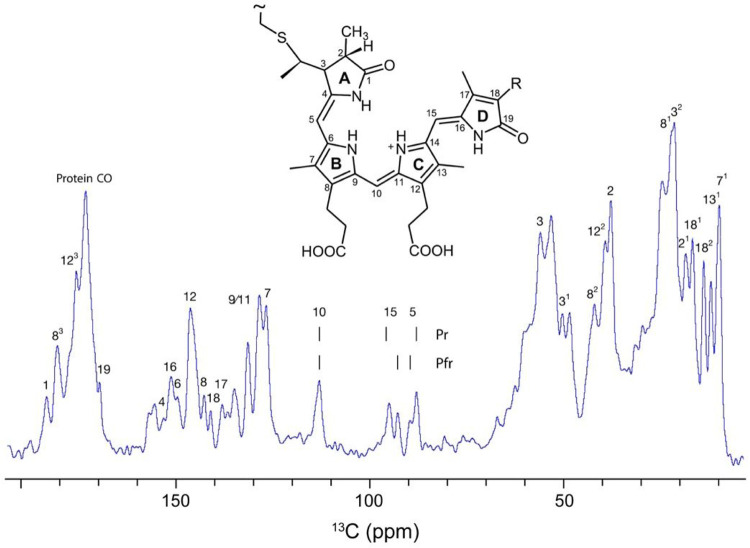
1D ^13^C NMR spectrum of the sedimented phytochrome Cph1 solely labeled in its chromophore (PCB, inset), recorded at 261 K and 20 kHz MAS at a field of 18.8 T.

A 1.0 mM Cph1 sample, with ^13^C, ^15^N labelling exclusively in the phycocyanobilin chromophore, was sedimented into a 1.9 mm rotor. The 1D ^13^C NMR spectrum showed well-resolved signals for methine carbons C5, C10, and C15 (inset of Fig. 7), at the expected chemical shift positions, and similar linewidths for all signals. Exchange broadening due to ring reorientation or other alterations in the double-bond network due to protonation/deprotonation events were not apparent (Fig. 7). In those measurements, the signal-to-noise ratio (S/N) was low necessitating several hours of acquisition, presumably due to considerable Brownian motion within the sample, diminishing the efficiency of the CP.

A similar sample of Cph1 but with amino-acid selective ^13^C, ^15^N labelling of Ile, Arg, Val and Trp was prepared for DNP measurements from a 1.7 mM solution, to which the radical BcTolM dissolved in d_8_-glycerol was added, so that the final glycerol concentration in the buffer was low (11% d_8_-glycerol/22% D_2_O/67% H_2_O; v/v). This sample showed substantial 20-fold Boltzmann enhancements (Fig. S12), enabling collection of complex 3D hCANcoCA spectra (Chevelkov et al. 2013) with sufficiently good S/N in 4.5 days at a ^1^H NMR frequency of 800 MHz. Here, the substantial Boltzmann enhancement at 105 K indicates that freeze concentration of polarizing agent was suppressed despite the low glycerol concentration.

### Concluding remarks

Here, we report parameters, procedures and tools for sedimenting proteins in a molecular-weight range between 40–140 kDa into MAS rotors in order to record well-resolved ^1^H-^15^N correlations and sets of 3D spectra. By means of the outlined procedure, a dense phase is produced whose NMR spectra do not show noticable heterogeneous broadening, thus exhibiting a liquid-like appearance. Yet, CP provides an efficient magnetization transfer mechanism. In case of *Sulf*ETF we showed that the water-to-protein-ratio is 1:2.5 (w/w) and remains stable over time, with very similar spectra obtained after one year. Structural integrity of the sedimented proteins was demonstrated by comparison of the CP-based ^1^H-^15^N correlation spectra recorded on the dense phases with solution ^1^H-^15^N HSQC spectra or CP-based correlations obtained from crystalline samples.

These dense phases of folded proteins are generated by ultracentrifugation. In LLPS, similar dense phases occur, yet of mostly unstructured proteins, following suitable changes of buffer conditions. Those dense phases form spontaneously after induction of LLPS and tend to develop rapidly towards fibrillar states, often involving short repeats in their amino acid sequence. In principle, one could expect such tendency to form fibrils to be a problem of our approach. However, the dense phases of well-folded, globular proteins produced by ultracentrifugation did not show signs of fibrils even after weeks, as demonstrated by the set of clean 3D spectra obtained on *St*TS (Fig. S7) or by the long-term *Sulf*ETF spectrum shown in Fig. S6.

Among the factors influencing sensitivity, lower temperature helps to increase S/N in CP-based experiments. ^1^H-^15^N correlation spectra obtained after sedimentation show overall good resolution in the proton dimension (150–300 Hz at 100 kHz MAS), and excellent resolution in the nitrogen dimension. The nitrogen line widths of 20–30 Hz observed in spectra of *Sulf*ETF already indicates that the use of TROP-like sequences that exploit both, dipolar and scalar couplings for C-N transfer may be advantageous (Blahut et al. 2022). Strikingly, such dense phases are suitable for DNP experiments at low glycerol concentration. At a magnetic field of 18.8 T Boltzmann enhancements in the range of 20 are obtained with glycerol concentrations as low as 11% v/v.

The investigation of protein sediments instead of crystalline preparations may be advantageous when additives are required for crystallization that cannot easily be deuterated or carbon-depleted. Furthermore, there is larger freedom in the choice of pH and other buffer conditions than typically required for crystallization. In practical cases the experimentalist is concerned with the tradeoff between the slightly larger line width observed in spectra of dense phases than in spectra of crystalline samples and the larger experimental freedom. Our approach also has advantages compared to solution NMR in that no deuteration of the protein is required when spinning the sample at 100 kHz MAS and above. A complete set of signals can thus be obtained. It is further of value when cells of higher organisms than bacteria or yeast are employed as expression hosts since complete deuteration cannot be achieved in this case.

The possibility of obtaining extended data sets with well-resolved spectra of sedimented proteins has far-reaching consequences regarding the current paradigm of NMR. Soluble proteins of intermediate size that were currently not easily tractable by solution NMR are now accessible with the help of MAS NMR methods. It was demonstrated by Wiegand et al. that protein-DNA and protein-RNA complexes remain stable under fast MAS conditions (Wiegand et al. 2020). Considering the special advantage that deuteration can be avoided and that non-exchangeable protons can aid assignment procedures and enrich functional studies, we expect a particularly high impact of the presented approach on the investigation of RNA and their complexes.

## Figures and Tables

**Table 1:** Parameters relevant for the sedimentation procedure. For each sample, the molecular weight, the outer diameter of the rotor that was filled and the concentration prior to the ultracentrifugation step are given in the first three columns, respectively. The centrifugation itself is characterized by the maximum relative gravitational force and temperature during the centrifugation together with the total centrifugation time. n.d.: not determined; Cph1-WIRV: Cph1 solely ^13^C, ^15^N-labeled in trp, ile, arg and val; Cph1-PCB: only the cofactor is ^13^C, ^15^N-labeled. *Pf*TrpB(2B9) bears the mutations I16V, E17G, I68V, F95L, F274S, T292S, T321A, V384A as compared to the wildtype.

Sample	Mol. weight (kDa)	Rotor (mm)	Conc. (μM)	Force (x1000g)	Temp. (K)	Centrifugation Time (h)
Cph1-WIRV	59	1.9	1700	71	278	67
Cph1-PCB	59	1.9	1000	71	278	68
FcRN	42	0.7	100	100	277	40
*StS*	144	0.7	140	71	281	42
SulfETF	67	0.7	n.d.	82	278	70
*Pf*TrpB	85	0.7	340	71	298	94
*Pf*TrpB(2B9)	85	1.3	450	210	298	72
